# Urinary bladder partial carbon dioxide tension during hemorrhagic shock and reperfusion: an observational study

**DOI:** 10.1186/cc3797

**Published:** 2005-08-17

**Authors:** Arnaldo Dubin, Mario O Pozo, Vanina S Kanoore Edul, Gastón Murias, Héctor S Canales, Marcelo Barán, Bernardo Maskin, Gonzalo Ferrara, Mercedes Laporte, Elisa Estenssoro

**Affiliations:** 1 Medical Director, Intensive Care Unit, Sanatorio Otamendi y Miroli, Buenos Aires, Argentina; 2 Staff physician, Intensive Care Unit, Clínicas Bazterrica y Santa Isabel, Buenos Aires, Argentina; 3 Research Fellow, Cátedra de Farmacología, Facultad de Ciencias Médicas, Universidad Nacional de La Plata, La Plata, Argentina; 4 Staff physician, Intensive Care Unit, Clínicas Bazterrica y Santa Isabel, Buenos Aires, Argentina; 5 Staff physician, Intensive Care Unit, Hospital San Martín de La Plata, Argentina; 6 Medical Director, Renal Transplantation Unit, CRAI Sur, CUCAIBA, Argentina; 7 Medical Director, Intensive Care Unit, Hospital Posadas, Buenos Aires, Argentina; 8 Resident, Intensive Care Unit, Hospital San Martín de La Plata, Argentina; 9 Medical Director, Clinical Chemistry Laboratory, Hospital San Martín de La Plata, Argentina; 10 Medical Director, Intensive Care Unit, Hospital San Martín de La Plata, Argentina

## Abstract

**Introduction:**

Continuous monitoring of bladder partial carbon dioxide tension (PCO_2_) using fibreoptic sensor technology may represent a useful means by which tissue perfusion may be monitored. In addition, its changes might parallel tonometric gut PCO_2_. Our hypothesis was that bladder PCO_2_, measured using saline tonometry, will be similar to ileal PCO_2 _during ischaemia and reperfusion.

**Method:**

Six anaesthetized and mechanically ventilated sheep were bled to a mean arterial blood pressure of 40 mmHg for 30 min (ischaemia). Then, blood was reinfused and measurements were repeated at 30 and 60 min (reperfusion). We measured systemic and gut oxygen delivery and consumption, lactate and various PCO_2 _gradients (urinary bladder–arterial, ileal–arterial, mixed venous–arterial and mesenteric venous–arterial). Both bladder and ileal PCO_2 _were measured using saline tonometry.

**Results:**

After bleeding systemic and intestinal oxygen supply dependency and lactic acidosis ensued, along with elevations in PCO_2 _gradients when compared with baseline values (all values in mmHg; bladder ΔPCO_2 _3 ± 3 versus 12 ± 5, ileal ΔPCO_2 _9 ± 5 versus 29 ± 16, mixed venous–arterial PCO_2 _5 ± 1 versus 13 ± 4, and mesenteric venous–arterial PCO_2 _4 ± 2 versus 14 ± 4; *P *< 0.05 versus basal for all). After blood reinfusion, PCO_2 _gradients returned to basal values except for bladder ΔPCO_2_, which remained at ischaemic levels (13 ± 7 mmHg).

**Conclusion:**

Tissue and venous hypercapnia are ubiquitous events during low flow states. Tonometric bladder PCO_2 _might be a useful indicator of tissue hypoperfusion. In addition, the observed persistence of bladder hypercapnia after blood reinfusion may identify a territory that is more susceptible to reperfusion injury. The greatest increase in PCO_2 _gradients occurred in gut mucosa. Moreover, the fact that ileal ΔPCO_2 _was greater than the mesenteric venous–arterial PCO_2 _suggests that tonometrically measured PCO_2 _reflects mucosal rather than transmural PCO_2_. Ileal ΔPCO_2 _appears to be the more sensitive marker of ischaemia.

## Introduction

Monitoring the adequacy of tissue oxygenation in critically ill patients is a challenging task [1]. Despite extensive research, tissue capnometry remains the only clinically relevant approach to monitoring regional perfusion and oxygenation. Elevation in tissue partial carbon dioxide tension (PCO_2_) might represent a better surrogate of hypoperfusion than other systemic and regional parameters [2,3].

During the past 20 years a large body of clinical evidence was developed supporting the usefulness of gastrointestinal PCO_2 _tonometry for the monitoring of tissue perfusion [4]. Gastric tonometry can readily be performed in the critically ill and gives significant information on outcomes [5,6]. It may also be a helpful guide in therapeutic decision making [7]. Nevertheless, technical difficulties and frequent artefacts have dampened the initial enthusiasm [8]. In an attempt to overcome the limitations of gastric tonometry, sublingual capnometry was then developed [9]. Despite initial interest and potential advantages, this technique has neither been completely validated nor widely used [10].

More recently, tissue perfusion has been assessed with continuous monitoring of bladder PCO_2 _using fibreoptic sensor technology [11,12], yielding interesting findings in experimental models of ischaemia/reperfusion. Although the equipment required may be expensive, bladder PCO_2 _can readily be measured via a urinary catheter incorporating a silicone balloon. Our goal in the present study was to compare bladder PCO_2 _measured using saline tonometry versus other tissue and venous PCO_2 _values. Our hypothesis was that bladder PCO_2 _will track ileal PCO_2 _during ischaemia and reperfusion.

## Materials and methods

### Surgical preparation

Six sheep were anaesthetized with 30 mg/kg sodium pentobarbital, intubated and mechanically ventilated (Harvard Apparatus Dual Phase Control Respirator Pump Ventilator; South Natick, MA, USA) with a tidal volume of 15 ml/kg, a fractional inspired oxygen of 0.21, and positive end-expiratory pressure adjusted to maintain arterial oxygen saturation above 90%. The respiratory rate was set to keep the end-tidal PCO_2 _at 35 mmHg. Neuromuscular blockade was applied with intravenous pancuronium bromide (0.06 mg/kg). Additional pentobarbital boluses (1 mg/kg per hour) were administered.

Catheters were advanced through the left femoral vein to administer fluids and drugs, and through left femoral artery to measure blood pressure and obtain blood gases. A pulmonary artery catheter was inserted through the right external jugular vein (Flow-directed thermodilution fibreoptic pulmonary artery catheter; Abbott Critical Care Systems, Mountain View, CA, USA).

An orogastric tube was inserted to allow drainage of gastric contents. Then, a midline laparotomy and splenectomy were performed. An electromagnetic flow probe was placed around the superior mesenteric artery to measure intestinal blood flow. A catheter was placed in the mesenteric vein through a small vein proximal to the gut to draw blood gases. Tonometers (TRIP Sigmoid Catheter; Tonometrics, Inc., Worcester, MA, USA) were inserted through small ileotomy and cystostomy to measure ileal and urinary bladder intramucosal PCO_2_. A second catheter was placed through the same cystostomy to drain urine. Finally, after careful haemostasis, the abdominal wall incision was closed.

### Measurements and derived calculations

Arterial, systemic, pulmonary and central venous pressures were measured using corresponding transducers (Statham P23 AA; Statham, Hato Rey, Puerto Rico). Cardiac output was measured by thermodilution with 5 ml saline solution at 0°C (HP OmniCare Model 24 A 10; Hewlett Packard, Andover, MA, USA). An average of three measurements taken randomly during the respiratory cycle was considered and was referenced to body weight to yield the cardiac output (Q). Intestinal blood flow was measured with the electromagnetic method (Spectramed Blood Flowmeter model SP 2202 B; Spectramed Inc., Oxnard, CA, USA) with *in vitro *calibrated transducers of 5–7 mm diameter (Blood Flowmeter Transducer; Spectramed Inc.). Occlusive zero was controlled before and after each experiment. Non-occlusive zero was corrected before each measurement. Superior mesenteric blood flow was referenced to gut weight (Q_intestinal_).

Arterial, mixed venous and mesenteric venous partial oxygen tension (PO_2_), PCO_2 _and pH were measured using a blood gas analyzer (ABL 5; Radiometer, Copenhagen, Denmark), and haemoglobin and oxygen saturation were measured using a co-oximeter calibrated for sheep blood (OSM 3; Radiometer). Arterial oxygen content (CaO_2_), mixed venous oxygen content (CvO_2_) and mesenteric venous oxygen content (CvmO_2_) were calculated as follows: haemoglobin × 1.34 × oxygen saturation + PO_2 _× 0.0031. Systemic and intestinal oxygen delivery (DO_2_) and oxygen consumption (VO_2_) were calculated as follows: systemic DO_2 _= Q × CaO_2_; systemic VO_2 _= Q × (CaO_2 _- CvO_2_); intestinal DO_2 _= Q_intestinal _× CaO_2_; and intestinal VO_2 _= Q_intestinal _× (CaO_2 _- CvmO_2_).

Arterial lactate concentration was measured using an automatic analyzer (Hitachi 912; Boehringer Mannheim Corporation, Indianapolis, IN, USA).

Bladder and ileal intramucosal PCO_2 _were measured using a tonometer filled with 2.5 ml saline solution. Of the solution, 1.0 ml was discarded after an equilibration period of 30 min, and PCO_2 _was measured in the remaining 1.5 ml. These values were corrected for the equilibration period and were used to calculate intramucosal-arterial gradients (bladder and ileal ΔPCO_2_). Mixed venous–arterial PCO_2 _(Pv–aCO_2_) and mesenteric venous–arterial PCO_2 _differences (Pvm–aCO_2_) were also calculated.

### Experimental procedure

Basal measurements were taken after a stabilization period longer than 30 min. Then, sheep were bled to a mean arterial blood pressure of 40 mmHg for 30 min (ischaemia). This degree of arterial hypotension was maintained by extracting or returning blood, as necessary. Collected blood was heparinized (5,000 U/l) and stored in a warmed water bath (37.5°C). Then, blood was reinfused and measurements were repeated at 30 and 60 min (reperfusion).

At the end of the experiment the animals were killed with an additional dose of pentobarbital and a KCl bolus. A catheter was inserted into the superior mesenteric artery and Indian ink was instilled. Dyed intestinal segments were dissected, washed and weighed to calculate gut indices.

The local Animal Care Committee approved the study. Care of animals was in accordance with US National Institute of Health guidelines.

### Statistical analysis

Data were assessed for normality and expressed as mean ± standard deviation. Differences were analyzed using repeated measures analysis of variance and Dunnett's multiple comparisons test to compare each time point with baseline. One-time comparisons between different PCO_2 _gradients were tested using one-way analysis of variance and Newman–Keuls multiple comparisons test.

## Results

### Haemodynamic and oxygen transport effects

Mean arterial pressure decreased during bleeding, as did Q, Q_intestinal _and systemic and intestinal DO_2 _and VO_2_. These variables returned to basal values after reinfusion of blood, with the exception of mean arterial pressure and systemic VO_2_, which remained higher than basal values (Table 1).

### Metabolic effects

Metabolic acidosis and hyperlactataemia developed during ischaemia, and persisted after reinfusion (Table 2).

### Effects on partial carbon dioxide tension gradients

**Figure 1:**
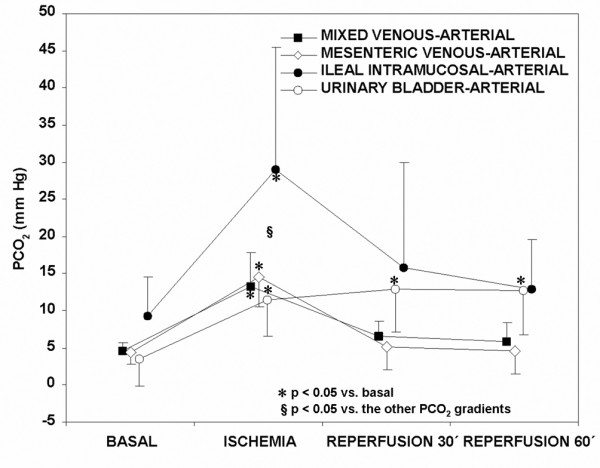
Behaviour of PCO_2 _gradients. Shown are the various partial carbon dioxide tension (PCO_2_) gradients in basal conditions, during ischaemia and after reperfusion.

Mixed and mesenteric venoarterial and urinary bladder and ileal ΔPCO_2 _differences increased during ischaemia. Ileal ΔPCO_2 _was higher than other PCO_2 _gradients during ischaemia (Fig. 1). The change in ileal ΔPCO_2 _(20 ± 10 mmHg) during ischaemia was greater than that in bladder ΔPCO_2 _(8 ± 7 mmHg) and in Pv–aCO_2 _(9 ± 5 mmHg) and Pvm–aCO_2 _(10 ± 3 mmHg; *P *< 0.05 for bladder ΔPCO_2_, Pv–aCO_2 _and Pvm–aCO_2 _versus ileal ΔPCO_2_). However, all PCO_2 _gradients returned to basal values after reperfusion, except for bladder ΔPCO_2_, which remained elevated (Fig. 1).

## Discussion

The main finding in the present study is the consistent expression of hypercapnia during low flow states. High PCO_2 _values were evident in veins, ileum and even urinary bladder. In contrast to the other carbon dioxide gradients, bladder ΔPCO_2 _remained elevated after reperfusion.

The prevention, detection and correction of tissue dysoxia are main goals in the management of critically ill patients [1]. Gastric tonometry has been considered the only available method to track tissue oxygenation in the clinical arena [1]. However, tissue hypercapnia is not just a marker of dysoxia but is also an indicator of hypoperfusion. Tissue and venous PCO_2 _remain unchanged in states of tissue dysoxia with preserved blood flow, such as hypoxic and anaemic hypoxia [13-15]. On the other hand, in a high flow state, such as sepsis, measurements of intramucosal acidosis remain helpful because of the frequent presence of microcirculatory derangements [16]. Moreover, increased blood flow may correct tissue hypercapnia in endotoxaemia [17].

Although most studies dealing with tissue capnometry have focused on the gastrointestinal tract, others have been performed in muscle [18,19], renal parenchyma [20,21] and subcutaneous tissue [22]. Few studies have assessed urinary PCO_2 _for the monitoring of tissue oxygenation. Lin and coworkers [23] measured urinary PCO_2 _in critically ill patients to evaluate the adequacy of perfusion. Urinary PCO_2 _was higher in shock than in control patients (79 ± 10 mmHg versus 43 ± 2 mmHg; *P *< 0.0001). Lang and colleagues [11] measured urinary bladder gases using a fibreoptic sensor in a swine model of ischaemia/reperfusion. After 30 min of aortic clamping bladder PCO_2 _increased from 57 ± 5 mmHg to 117 ± 7 mmHg, and it returned to baseline after 60 min of reperfusion. Clavijo-Alvarez and coworkers [12] studied this issue in a model of haemorrhagic shock in which pigs were bled and kept at a mean arterial pressure of 40 mmHg until decompensation. Animals were then resuscitated with shed blood plus lactated Ringer's solution and observed for 2 hours. In contrast to our findings, those investigators found greater increases in bladder PCO_2_; basal PCO_2 _was 49 ± 6 mmHg and increased to 71 ± 7 mmHg at the end of shock. Jejunal intramucosal PCO_2 _exhibited similar behaviour.

These differences might be related to the use of different animal species but also, and primarily, to the longer period of shock. Because the pigs in the study by Clavijo-Alvarez and coworkers [12] reached a lower cardiac output than did the sheep in our study, changes in surrogates of hypoperfusion such as base excess bicarbonate and bladder PCO_2 _were more pronounced. Nevertheless, gut intramucosal acidosis was similar in both studies, which might be related to the greater vulnerability of sheep intestinal mucosa to hypoperfusion. In addition, differences might be explained by diverse surgical preparations and methods for measuring intramucosal PCO_2_. Clavijo-Alvarez and coworkers completely isolated the bladder, and the PCO_2 _sensor was encased within the mucosa so that they could avoid interference. In this way, the measurements should reflect those from the bladder wall more accurately. Furthermore, they used a more sensitive method to measure PCO_2_. Nevertheless, it is difficult to reproduce this type of measurement in patients, and our methodology seems more suitable for clinical application.

Although tissue and venous hypercapnia is a widespread consequence of hypoperfusion, our experiments reveal that the increase in PCO_2 _is higher in ileal mucosa than in bladder mucosa and mixed and mesenteric venous blood. The underlying mechanism producing this preferential elevation in ileal ΔPCO_2 _might be related to particular characteristics of villi microcirculation. Countercurrent circulation might induce a functional shunt that could place distal microvilli segments at ischaemic risk [24]. There is some controversy regarding the meaning of intramucosal PCO_2_; specifically, does it reflect whole wall or superficial mucosal perfusion? An ileal ΔPCO_2 _greater than the Pvm-aCO_2 _suggests that tonometric PCO_2 _reflects mucosal rather than transmural PCO_2_. On the other hand, the similar increase in bladder–arterial and systemic and intestinal venoarterial PCO_2 _gradients suggests the presence of similar degrees of hypoperfusion. As previously described [25], the fraction of cardiac output directed to gut (superior mesenteric artery blood flow/cardiac output) decreased during ischaemia (from 0.23 ± 0.06 to 0.16 ± 0.07; data not shown). However, this was not enough to produce differences between systemic and intestinal venoarterial PCO_2 _gradients.

Another interesting finding of this study lies in the persistence of bladder intramucosal acidosis during reperfusion. Recent studies indicated that ischaemia/reperfusion can cause acute inflammation and contractile dysfunction of the bladder [26]. Bajory and coworkers [27] demonstrated severe microcirculatory derangements such as decreased functional capillary density, red blood cell velocity, venular and arteriolar diameter, and enhanced macromolecular leakage after bladder ischaemia/reperfusion. We speculate that these microcirculatory alterations might lead to decreased carbon dioxide removal. Again, differential susceptibility to injury between species could explain differences from other studies [11,12].

Limitations of the present study could be related to the method of measurement of bladder PCO_2_. First, tonometric measurement of PCO_2 _has drawbacks [8]. Second, urine itself could potentially influence tonometric PCO_2 _beyond perfusion deficits. In fact, urine can have variable carbon dioxide content, resulting, for example, from different grades of carbonic anhydrase inhibition or from systemic bicarbonate administration [28]. Actually, failure to observe an appropriate increase in urinary-blood PCO_2 _during bicarbonate loading has been employed as an index of reduced distal nephron proton secretion in distal renal tubular acidosis [28]. Changes in systemic oxygenation can also modify urine composition. Moriguchi and coworkers [29] have showed that urinary bicarbonate, calculated from urinary PCO_2 _and pH, increases after anaerobic exercise. Those authors related these findings to systemic carbon dioxide production and later urinary excretion [29]. They also described a circadian rhythm in urinary bicarbonate elimination [30]. Moreover, an elevated bladder ΔPCO_2 _could also represent a late manifestation of renal hypoperfusion. Further studies are needed to clarify the influence of renal carbon dioxide excretion on bladder PCO_2_.

## Conclusion

Our data suggest that bladder ΔPCO_2 _could be a useful indicator of tissue perfusion. However, intestinal ΔPCO_2 _is the more sensitive carbon dioxide gradient for monitoring low flow states. Further studies are needed to establish the definitive monitoring value of urinary PCO_2_.

## Key messages

• 	Urinary bladder ΔPCO_2 _may be a useful indicator of tissue perfusion, but intestinal ΔPCO_2 _is the more sensitive carbon dioxide gradient for the monitoring of low flow states.

• 	The fact that the observed ileal ΔPCO_2 _was greater than Pvm-aCO_2 _suggests that tonometric PCO_2 _reflects mucosal rather than transmural PCO_2_.

## Abbreviations

CaO_2 _= arterial oxygen content; CvmO_2 _= mesenteric venous oxygen content; CvO_2 _= mixed venous oxygen content; DO_2 _= oxygen transport; PCO_2 _= partial carbon dioxide tension; PO_2 _= partial oxygen tension; Pv–aCO_2 _= mixed venous-arterial PCO_2 _difference; Pvm–aCO_2 _= mesenteric venous–arterial PCO_2 _difference; Q = cardiac output; VO_2 _= oxygen consumption.

## Competing interests

The author(s) declare that they have no competing interests.

## Authors' contributions

AD was responsible for the study concept and design, analysis and interpretation of data, and drafting of the manuscript. MOP, VSKE, GM and HSC performed acquisition of data and contributed to drafting of the manuscript. BM and ML conducted blood determinations and contributed to drafting of the manuscript. MB and GF performed the surgical preparation and contributed to the discussion. EE helped in the drafting of the manuscript and conducted a critical revision for important intellectual content. All authors read and approved the final manuscript.

## Figures and Tables

**Table 1:** Haemodynamic and oxygen transport parameters at basal conditions, during ischaemia, and after 30 and 60 min of reperfusion

			Reperfusion	
			
Parameter	Basal	Ischemia	30 min	60 min
Mean arterial blood pressure (mmHg)	87 ± 14	38 ± 4	105 ± 10*	104 ± 10*
Cardiac output (ml/min per kg)	138 ± 10	70 ± 17*	136 ± 17	137 ± 16
Intestinal blood flow (ml/min per kg)	787 ± 181	272 ± 100*	890 ± 278	756 ± 134
Systemic oxygen transport (ml/min per kg)	19.5 ± 2.7	7.8 ± 1.9*	18.8 ± 2.8	19.3 ± 3.2
Systemic oxygen consumption (ml/min per kg)	6.8 ± 1.0	5.7 ± 1.5*	7.4 ± 1.2*	7.2 ± 0.9*
Systemic oxygen extraction ratio	0.35 ± 0.06	0.72 ± 0.08*	0.40 ± 0.09*	0.39 ± 0.09
Intestinal oxygen transport (ml/min per kg)	112.5 ± 35.2	31.1 ± 14.0*	126.1 ± 51.1	107.8 ± 28.7
Intestinal oxygen consumption (ml/min per kg)	30.3 ± 4.6	19.3 ± 7.1*	31.3 ± 6.9	31.5 ± 6.6
Intestinal oxygen extraction ratio	0.29 ± 0.09	0.65 ± 0.12*	0.28 ± 0.11	0.31 ± 0.10

*P < 0.05 versus basal.

**Table 2:** Arterial, mixed venous and mesenteric venous blood gases, and arterial lactate at basal conditions, during ischemia and after 30 and 60 minutes of reperfusion

			Reperfusion	
			
Parameter	Basal	Ischaemia	30 min	60 min
Arterial pH	7.37 ± 0.03	7.36 ± 0.05	7.33 ± 0.05*	7.36 ± 0.04
Arterial PCO_2 _(mmHg)	38 ± 4	35 ± 5*	36 ± 4	36 ± 5
Arterial PO_2 _(mmHg)	77 ± 9	80 ± 15	75 ± 10	78 ± 8
Arterial HCO_3_^- ^(mmol/l)	22 ± 3	19 ± 2*	19 ± 2*	20 ± 2*
Arterial base excess (mmol/l)	-3 ± 3	-5 ± 2*	-6 ± 2*	-4 ± 3*
Mixed venous pH	7.34 ± 0.03	7.26 ± 0.03*	7.28 ± 0.04*	7.32 ± 0.04
Mixed venous PCO_2 _(mmHg)	43 ± 4	48 ± 5*	43 ± 4	42 ± 3
Mixed venous PO_2 _(mmHg)	38 ± 4	23 ± 3*	37 ± 4	39 ± 4
Mixed venous HCO_3_^- ^(mmol/l)	23 ± 3	21 ± 3	20 ± 2	21 ± 2
Mixed venous base excess (mmol/l)	-3 ± 3	-6 ± 3*	-7 ± 2*	-5 ± 2*
Mesenteric venous pH	7.34 ± 0.03	7.26 ± 0.03*	7.30 ± 0.05*	7.32 ± 0.04
Mesenteric venous PCO_2 _(mmHg)	42 ± 5	49 ± 5*	41 ± 4	41 ± 4
Mesenteric venous PO_2 _(mmHg)	43 ± 7	26 ± 3*	42 ± 6	43 ± 5
Mesenteric venous HCO_3_^- ^(mmol/l)	23 ± 3	22 ± 2	20 ± 2*	21 ± 2
Mesenteric venous base excess (mmol/l)	-3 ± 3	-5 ± 2*	-6 ± 2*	-5 ± 2*
Arterial lactate (mmol/l)	1.6 ± 0.5	3.7 ± 1.7*	3.9 ± 2.0*	3.2 ± 1.5*

Values are expressed as mean ± standard deviation. *P < 0.05 versus basal. PCO_2_, partial carbon dioxide tension; PO_2_, partial oxygen tension.
